# Potent SARS-CoV-2-Specific T Cell Immunity and Low Anaphylatoxin Levels Correlate With Mild Disease Progression in COVID-19 Patients

**DOI:** 10.3389/fimmu.2021.684014

**Published:** 2021-06-14

**Authors:** Eliott Lafon, Gabriel Diem, Christina Witting, Viktoria Zaderer, Rosa Maria Bellmann-Weiler, Markus Reindl, Angelika Bauer, Andrea Griesmacher, Vilmos Fux, Gregor Hoermann, Carl Miller, August Zabernigg, Ewald Wöll, Doris Wilflingseder, Cornelia Lass-Flörl, Wilfried Posch

**Affiliations:** ^1^ Institute of Hygiene and Medical Microbiology, Medical University of Innsbruck, Innsbruck, Austria; ^2^ University Hospital of Internal Medicine II, Medical University of Innsbruck, Innsbruck, Austria; ^3^ Clinical Department of Neurology, Medical University of Innsbruck, Innsbruck, Austria; ^4^ Central Institute for Medical and Chemical Laboratory Diagnosis, University Hospital Innsbruck, Innsbruck, Austria; ^5^ Munich Leukemia Laboratory (MLL), Munich, Germany; ^6^ Department of Internal Medicine, Hospital Kufstein, Kufstein, Austria; ^7^ Department of Internal Medicine, Hospital Zams, Zams, Austria

**Keywords:** SARS-CoV-2, T cell immunity, neutralizing Abs, anaphylatoxin, ELISpot assay, COVID-19

## Abstract

T cells play a fundamental role in the early control and clearance of many viral infections of the respiratory system. In SARS-CoV-2-infected individuals, lymphopenia with drastically reduced CD4^+^ and CD8^+^ T cells correlates with Coronavirus disease 2019 (COVID-19)-associated disease severity and mortality. In this study, we characterized cellular and humoral immune responses induced in patients with mild, severe and critical COVID-19. Peripheral blood mononuclear cells of 37 patients with mild, severe and critical COVID-19 and 10 healthy individuals were analyzed by IFNγ ELISpot and multi-color flow cytometry upon stimulation with peptide pools covering complete immunodominant SARS-CoV-2 matrix, nucleocapsid and spike proteins. In addition SARS-CoV-2 antibody levels, neutralization abilities and anaphylatoxin levels were evaluated by various commercially available ELISA platforms. Our data clearly demonstrates a significantly stronger induction of SARS-CoV-2 specific CD8^+^ T lymphocytes and higher IFNγ production in patients with mild compared to patients with severe or critical COVID-19. In all patients SARS-CoV-2-specific antibodies with similar neutralizing activity were detected, but highest titers of total IgGs were observed in critical patients. Finally, elevated anaphylatoxin C3a and C5a levels were identified in severe and critical COVID-19 patients probably caused by aberrant immune complex formation due to elevated antibody titers in these patients. Crucially, we provide a full picture of cellular and humoral immune responses of COVID-19 patients and prove that robust polyfunctional CD8^+^ T cell responses concomitant with low anaphylatoxin levels correlate with mild infections. In addition, our data indicates that high SARS-CoV-2 antibody titers are associated with severe disease progression.

## Introduction

At the end of 2019 a novel betacoronavirus that causes a severe respiratory syndrome in humans was discovered in Wuhan City, Hubei Province, China—subsequently the virus was called Severe Acute Respiratory Syndrome Coronavirus 2 (SARS-CoV-2) (1–3). SARS-CoV-2 is the causative agent for the Coronavirus Disease 2019 (COVID-19) and is also responsible for the current global pandemic, which was announced by the World Health Organization on the 13th of March 2020 (WHO). The most common symptoms are fever, dry cough and tiredness and sometimes also include headache, sore throat, dyspnea, diarrhea, loss of taste and smell or myalgia. Furthermore, in critical cases, patients can develop severe pneumonia, abnormalities of granulocytes and monocytes, increased cytokine production and acute respiratory distress syndrome (ARDS) which can be fatal (4, 5). In addition, studies revealed that approximately 15.6% of SARS-CoV-2 infections are asymptomatic, but their contribution to silent transmission of the virus is currently controversially discussed (6–10).

According to studies on SARS-CoV, recovered patients produced virus-specific IgG antibodies and neutralizing antibodies after infection, which started to wane after 16 months and after 3 years only 50% of convalescent patients still had SARS-CoV-specific IgGs (11). In contrast, Oi-Wing and colleagues showed that even after 11 years post-infection, SARS-CoV-specific memory T cells were still found to persist in SARS-recovered individuals, indicating a long lasting T cell immunity (12). For COVID-19, data also illustrated that IgM and IgG could be detected as early as 3 and 7 days respectively post-disease onset and could remain for at least 6–8 months but additional longitudinal serological studies, that follow patients’ immunity over an extended period, are needed (13, 14). Other studies however highlight a decrease of IgG and neutralizing antibodies in SARS-CoV-2 recovered individuals within 2–3-month post infection and some data even expose the possibility of re-infection with SARS-CoV-2, leading to milder or more severe outcome. These variations probably depending on the degree of immunity achieved after the first infection (15–17). Of note, reports show that the titers of neutralizing antibody seem to be variable among patients and sometimes even drop below detection limit, while Ni et al. did not detect impact on SARS-COV-2-specific T cell immunity (14, 18). In this context, using multiple experimental approaches, SARS-CoV-2-specific CD4^+^ and CD8^+^ T lymphocytes against structural and non-structural regions were observed in all COVID-19 cases, even among antibody-seronegative asymptomatic and mild COVID-19 cases (19, 20). In fact, total lymphocytes, CD4^+^ T cells, CD8^+^ T cells and CD4^+^/CD8^+^ T cell ratios were investigated and higher cell numbers correlated with the inflammatory status of the patient (21). Importantly, CD8^+^ T cell were shown to play a critical role in mediating viral clearance and also to represent a reliable indicator of disease severity and recovery (21). In addition, IFNγ production of T cells, especially CD4^+^ T cells, were associated with mild or moderate disease progression (22).

In patients with severe or critical COVID-19 uncontrolled inflammatory innate immune responses have been reported, which were associated with impaired adaptive immunity and harmful tissue damage (23). Liao et al. characterized bronchoalveolar lavage fluid immune cells from patients with severe COVID-19 and found impaired numbers of expanded CD8^+^ T cells and abundant numbers of monocyte-derived macrophages as a cause for cytokine overexpression and disease severity (24). We and others identified a link between complement regulation and cytokine overexpression in SARS-CoV-2 infected tissues, since complement deposition and high anaphylatoxin serum levels have been reported in patients with severe or critical disease (25). Also in murine models it has been previously shown that, during infection with different human coronaviruses, depletion or blockage of complement component 3 (C3) in SARS-CoV infection and complement component 5 (C5) in MERS-CoV infection resulted in milder disease severity (26, 27).

In this study, we characterized cellular and humoral immune responses induced in patients with mild, severe and critical COVID-19. For this, we characterized the T cell immunity from 37 patients with mild, severe and critical COVID-19 as well as 10 healthy donors. Total SARS-CoV-2-specific T cells and virus-specific CD4^+^ and CD8^+^ T cells were assessed dependent on their ability to recognize matrix (M), nucleocapsid (N) or spike (S) protein of SARS-CoV-2 by IFNγ ELISpot or multi-color flow cytometry. Furthermore, SARS-CoV-2-specific antibody titers, neutralization capacity and IC50 values from neutralization curves of plasma samples were determined. Finally, expression of anaphylatoxins C3a and C5a of patients with mild, severe and critical COVID-19 were analyzed. Together, our results indicate that early and polyfunctional CD8^+^ T cell immunity along with low anaphylatoxin expression is associated with viral clearance and mild to moderate disease progression.

## Methods

### Ethics Statement

Written informed consent was obtained from all donors of leftover nasopharyngeal/oropharyngeal specimens, EDTA blood and Serum samples by the participating clinics. The Ethics Committee of the Medical University of Innsbruck approved the use of anonymized leftover specimens of COVID-19 patients (ECS1166/2020) and healthy donors (ECS1166/2018) for scientific purposes.

### Human Samples

In this study 37 COVID-19 patients with mild (n = 11), severe (n = 15) or critical (n = 11) disease progression were included. According to the clinical data available patients were divided into three groups based on ECDC guidelines on disease severity. The median age of all patients was 66 years (28–88 years) and the percentage of female and male patients included in the study was 34.4 and 65.6% respectively (**Table 1**). The average sampling day of analyzed samples, defined as the time (days) between positive SARS-CoV-2 PCR testing and blood sampling, was 37 days (18–61 days) (**Table 1**). In addition, frozen PBMCs from 10 healthy donors collected before the COVID-19 pandemic (before October 2019) were used as negative controls.

**Table 1:** Characteristics from patients included in the study by COVID-19 severity (mild, severe, critical).

ID	Severity	Gender	Age	Symptoms	Underlying disease	ICU	Sampling day
1	Mild	M	65	Cough, Fever, Headache	alcohol abuse, CHD, arteriosclerosis	N	40
20	Mild	M	49	Cough, Fatigue	None	N	33
27	Mild	F	34	Cough, Fever	None	N	29
28	Mild	F	28	Mid abdominal pain	None	N	31
37	Mild	F	75	Fever, Headache	None	N	46
54	Mild	M	71	Cough, Dyspnoea, Fever	Prostatic hyperplasia, hypercholesterolemia	N	18
55	Mild	F	69	Cough, Dyspnoea, Myalgia	Chronic bronchitis, laryngitis, right pneumonia, obstruction of small respiratory tract, steatosis hepatis, prediabetes	N	21
65	Mild	M	52	Fever, Dyspnoea, Dry cough, Fatigue, Headache, Diarrhea, Myalgia, Loss of taste and smell	None	N	25
66	Mild	F	45	Fever, Dyspnoea, Dry cough, Fatigue, Headache, Diarrhea, Myalgia	Influenza A 4 weeks before diagnostic	N	21
79	Mild	M	58	Fever, Dyspnoea, Dry cough, Fatigue, Headache, Diarrhea, Myalgia, Loss of taste and smell, Vertigo	None	N	48
80	Mild	F	53	Fever, Fatigue, Headache, Myalgia, Loss of taste and smell, Sore throat	None	N	62
n = 11		Female / Male	Mean				Mean
		45.5% / 54.5%	54.5				34.0
ID	Severity	Gender	Age	Symptoms	Underlying disease	ICU	Sampling day
2	Severe	M	71	Lower gastroint. bleeding, viral pneumonia	Art. hypertension, aortic thrombosis, obesity	Y	42
3	Severe	M	66	Cough, Dyspnoae, Fever	Art. hypertension, obesity	Y	44
7	Severe	M	43	Cough, Dyspnoea	Global respiratory failure with intubation, delirium after ICU, RIFLE II after acute kidney failure , obesity, prediabetes, steatosis hepatis, tinea inguinalis, suspicion of obstructive sleep apnea syndrome, suspicion of small-fiber PNP, urosepsis	Y	38
13	Severe	M	78	Fever, Dry cough, Fatigue,	exacerbated COPD IV, hypercholesterolemia, axial hiatal hernia	N	35
15	Severe	M	72	Reduced quality of consciousness, Dry cough, Weakness	Alcohol dependence with delirium tremens, liver cirrhosis, thrombopenia, prolonged QTc time after haloperidol iv, reflux oesophagitis, mixed intoxication alcohol and BDZ, Malory-Weiss crack, Wernicke encephalopathy	N	20
21	Severe	F	78	Cough, Fever, Sore throat	None	Y	28
22	Severe	M	63	Cough, Fever, Sore throat	Hypotonia, cystitis, SR alternating with coronary sinus rhythm, permanent urinary bladder drainage	Y	44
23	Severe	M	77		Renal insufficiency, hypertension, urosepsis	Y	26
25	Severe	M	77	Cough, Dyspnoea, Fever, Loss of apetite, Myalgia	Atrial fibrillation, hypertension	Y	35
26	Severe	M	71	Fever	None	Y	40
43	Severe	F	66	Radiation pneumonitis bilateral	Hypertension	N	45
44	Severe	F	88	NA	Art. Hypertension	N	43
48	Severe	F	62	Dyspnoea	Septic shock, Multiple organ failure (lung, kidney, lactic acidosis), CVVHDF since 3.4.20, hypertension, Borreliosis in 2018	Y	28
50	Severe	M	59	Fever, Dyspnoea, Dry cough, Shortness of breath, Diarrhea, Myalgia	Hypertension, depression	Y	61
57	Severe	F	69	Cough, Dyspnoea, Headache, Tiredness	Obesity, hypothyroidism, itrahepatic cholestasis in papillary sclerosis, symptomatic cavernoma highly parientally re-associated with DVA	N	22
**n = 15**		**Female / Male**	**Mean**			** **	**Mean**
		33.3% / 66.7%	69.3				36.7
ID	Severity	Gender	Age	Symptoms	Underlying disease	ICU	Sampling day
10	Critical	F	65	Cough with blood contamination, Dyspnoea, Fever, Myalgie	Obesity, appendectomy, initial diagnosis of diabetes mellitus, acute kidney failure KDIGO III, ICU delirium	Y	30
11	Critical	M	47	Cough, Fever, Weakness, Diarrhea	Obesity	Y	41
12	Critical	F	78	Cough, Chills, Dizziness, Headache, Myalgia	Ascending great saphenous vein phlebitis left, initial diagnosis of bilateral pulmonary embolism, acute kidney failure in the form of infections, intermittent, CVVH, left jugular vein thrombosis, aortic stenosis	Y	24
16	Critical	M	58	Fever, Dyspnoea, Dry cough, Sever myalgia	First manifestation of diabetes mellitus March 2020	Y	37
17	Critical	F	71	Fever, Dyspnoea, Myalgia, Swallowing problems, Difficult speaking	Bulbar mysthenia gravis, myasthenic crisis requiring intubation, osteoporosis, hysterectomy, appendectomy, substituted hypothyroidism, fructose / lactose intolerance	Y	56
24	Critical	M	76	Cough, Fever, Fatigue, Diarrhea, Loss of appetite, Sore throat	Hypotonia, cystitis, SR alternating with coronary sinus rhythm, permanent urinary bladder drainage	Y	44
38	Critical	M	73	Cough, Fever, Dyspnoea	Treated with Ebetrexat, hypertension, atrial flutter	Y	47
39	Critical	M	79	Reduced quality of consciousness, Fever, Dry cough,	Immunological bronchiolitis with long-term steroid therapy, hypercholesterolemia, chronic renal insufficiency, mild sensory axonal polyneuropahtia, bacterial superinfection	Y	46
40	Critical	M	64	Cough, Severe Dyspnoea, Fever, Myalgie	Axial hiatal hernia, hepatopathy, right renal cyst, prostate hypertrophy, nodular goiter, superficial carotid sclerosis, GERD	Y	52
41	Critical	M	53	Severe Dyspnoea	Traumatic splenectomy 2000	Y	42
42	Critical	F	61	Fever, Cold symptoms	CAG 2011, swallowing disorder, obesity	Y	52
**n = 11**		**Female / Male**	**Mean**				**Mean**
** **	** **	**36.4% / 63.6%**	**65.9**				**42.8**

About 37 patients diagnosed with COVID-19 were included in the study. Patients were divided into three groups according to disease course, mild (n = 11), severe (n = 15) and critical (n = 11). Severity was assessed using ECDC guidelines. Patients characteristics, symptoms, clinical information and sampling days are presented.

### Cell Isolation and Culture

EDTA-whole blood samples from COVID-19 hospitalized patients (n = 37) were collected and peripheral blood mononuclear cells (PBMCs) were isolated using BD PharmLyse^®^ Red Blood Cell lysis buffer according to manufacturer protocol (BD Biosciences, New Jersey, USA). Isolated PBMCs were either cultured and analyzed or cryopreserved until used in the assays. PBMCs were cultured and expanded for 5–7 days in RPMI (Sigma Aldrich, Missouri, USA), 10% AB serum, 1% L-Glutamine (Sigma Aldrich, Missouri, USA) containing 20 U/ml Interleukin-2 (Miltenyi Biotec, Bergisch Gladbach, Germany) and 2.5 µg/ml Phytohemagglutinin (Sigma Aldrich, Missouri, USA) before stimulation with peptide pool or cell stimulation cocktail.

### Viruses

Clinical specimens from COVID-19 positive swabs (Ethics statement, ECS1166/2020) and SARS-CoV-2 viruses from repositories (BEI Resources, Manassas, VA, USA; CFAR/NIBSC; Nr-52281, Nr-52282, NR-52286) were propagated according to the manufacturer’s instructions and used subsequently in neutralization assays.

### IFNγ Elispot

IFNγ production of activated PBMCs was assessed by enzyme-linked immunospot (ELISpot) assays. Briefly, ELISpot MultiScreen^®^_HTS_ 96-well Filter Plates (Millipore, Massachussetts, USA) were activated using 35% ethanol, washed and coated overnight with anti-human IFNγ mAb 1-D1K (2 µg/ml; Mabtech, Stockholm, Sweden). On the day of the analyzes, coating solution was removed and plates were saturated with D-PBS (Sigma Aldrich, Missouri, USA) containing 10% FCS (Sigma Aldrich, Missouri, USA) for 2 h at room temperature (RT). Some 5 × 10^5^ PBMCs/well were counted and seeded in RPMI supplemented with 5% AB serum and 1% L-Glutamine (all obtained from Sigma Aldrich, Missouri, USA). Cells were stimulated with 0.6 nM/ml PepTivator^®^ SARS-CoV-2 Peptide Pools (Miltenyi Biotec, Bergisch Gladbach, Germany) of either SARS-CoV-2 membrane glycoprotein (M), nucleocapsid phosphoprotein (N) or spike glycoprotein (S) in duplicates. As positive controls, cells were stimulated with a mixture of CMV/CEF/CEFTA peptide pools (2 µg/ml; Mabtech, Stockholm, Sweden) or PMA/ionomycin cell activation cocktail (1:500; Biolegend, San Diego, USA) in duplicates. To determine the background level for each donor, PBMCs were seeded with culture medium only. After overnight incubation at 37°C and 5% CO_2_ cells were removed and IFNγ production was revealed using biotinylated anti-human IFNγ mAb 7-B6-1 (1 µg/ml in D-PBS containing 0.5% FCS; Mabtech, Stockholm, Sweden) for 2 h at RT, followed by incubation of Streptavidin-Alkaline Phosphatase (1:1,000 in D-PBS containing 0.5% FCS; Sigma Aldrich, Missouri, USA), and finally treatment with 50 µl ready-to-use BCIP^®^/NBT Liquid substrate (Sigma Aldrich, Missouri, USA). After each step plates were washed five times with D-PBS. Emerged spots were counted using a ImmunoSpot analyzer (Cellular Technology Limited, Shaker Heights, Ohio, USA) and spot quality was checked using the ImmunoSpot Software v5.0.9.15.

### Flow Cytometry

SARS-CoV-2-specific activation was additionally investigated by flow cytometry using a BD FACSVerse analyzer (BD Biosciences, New Jersey, USA). For this, cytokine production of CD4^+^ and CD8^+^ T cells from patient PBMCs were investigated after stimulation to assess the level of IL-2, IFNγ, TNF-α (all obtained from Biolegend, San Diego, USA) and MIP1-β (BD Biosciences, New Jersey, USA). Some 5 × 10^5^ cells PBMCs of COVID-19 patients were exposed to various peptide pools or cell activator cocktail and incubated for 6 h at 37°C and 5% CO_2_. After 2 h of incubation Brefeldin A (1:1,000; Biolegend, San Diego, USA) was added to inhibit protein transport and thus cytokine secretion. Cells were stained with a viability dye (Ghost DyeTM Violet 510, Tonbo Biosciences, San Diego, USA) for 30 min at 4°C protected from light, before extracellular staining was performed using monoclonal antibodies against human CD4^+^ (Biolegend, San Diego, USA) and CD8^+^ (BD Biosciences, New Jersey, USA). After another incubation 30 min at 4°C protected from light, cells were washed and fixed with Intracellular Staining Fixation Buffer for 20 min at RT followed by permeabilization using Intracellular Staining Permeabilization Wash Buffer according to the manufacturer’s instructions (Biolegend, San Diego, USA). Intracellular staining was performed using monoclonal antibodies against human IL-2, IFN-γ, TNF-α (Biolegend, San Diego, USA) and MIP1-β (BD Biosciences, New Jersey, USA) diluted in Intracellular Staining Permeabilization Wash Buffer for 30 min at RT protected from light. Data were analyzed using BD FACSUITE v1.0.6 and BD FACS Diva v9.0 software (BD Biosciences, New Jersey, USA).

### Elisa/Eclia

Serum and Plasma samples from COVID-19 patients were retrieved from EDTA blood samples by centrifugation at 300*g* for 5 min and serum or plasma fractions were carefully collected and stored at −80°C until use. Patient samples were analyzed by ELISA and ECLIA to assess SARS-CoV-2-specific IgG titers by two commercially available tests; Anti-SARS-CoV-2 ELISA IgG (Euroimmun, Lübeck, Germany) and ElectroChemiLuminescence–Immunoassay (ECLIA) Elecsys^®^ Anti-SARS-CoV-2 (Roche, Basel, Switzerland). SARS-CoV-2 ELISA IgG test detects IgG antibodies against the S protein of SARS-CoV-2. For this test calibrators, controls as well as diluted patient samples were transferred in microplate wells according to pipetting protocol and incubated for 60 min at 37°C. The wells were washed three times using working-strength wash buffer and incubated with peroxidase-labelled anti-human IgG 30 min at 37°C. Wells were washed again and incubated with substrate solution for 30 min at RT followed by addition of stop solution. Photometric measurement was made at 450 nm. For the automated Elecsys^®^ Anti-SARS-CoV-2, which detects IgG antibodies against the N protein of SARS-CoV-2, patient samples were run on a Cobas 8800 System (Roche, Basel, Switzerland) according to the manufacturer’s instructions. Briefly, patient serum or plasma was incubated with a mix of biotinylated and ruthenylated nucleocapsid antigen, which caused double-antigen sandwich immune complexes in the presence of corresponding antibodies. After addition of streptavidin-coated microparticles, the reagent mixture was transferred to the measuring cell and by applying a voltage emitted signals were measured by a photomultiplier. Serum and plasma samples which were tested negative for IgG by ELISA and ECLIA were additionally tested for anti-SARS-CoV-2 IgA and IgM using the commercially available WANTAI SARS-CoV-2 Ab ELISA (Beijing Wantai Biological, Beijing, China). In brief, positive and negative controls as well as specimens are incubated in microwell plate for 30 min at 37°C. Wells are washed five times with wash buffer and wells let 30–60 s to soak. Chromogen solution A and chromogen solution B are mixed 1:1 into each well and incubated for 15 min at 37°C protected from light. After reaction was stopped using stop solution, absorbance was analyzed at 450 nm using a TECAN SUNRICE microplate reader (TECAN, Männedorf, Switzerland).

### Neutralization Plaque Assay

VeroE6/TMPRSS2 cells (1.8 × 10^5^) were seeded in a 24-well plate with culture medium (DMEM high glucose medium supplemented with 10% FCS, 1% L-Glutamine, 1% Penicillin/Streptomycin; all reagents were obtained from Sigma Aldrich, Missouri, USA) and incubated overnight at 37°C and 5% CO_2_. On the following day, whole serum or plasma samples were serial-diluted from 1:1 to 1:6,250 and incubated with SARS-CoV-2 strains (1.5 × 10^4^ PFU/ml) for 1 h at 37°C. After incubation serum/plasma-virus mix was ultracentrifuged and resuspended in DMEM high glucose medium supplemented with 5% FCS, 1% L-Glutamine, 1% Penicillin/Streptomycin. VeroE6/TMPRSS2 cells were then inoculated with antibody-opsonized SARS-CoV-2 for 30 min on a shaker at RT and 30 min in an incubator at 37°C and 5% CO_2_. After incubation, inoculation medium was replaced with culture medium containing 1.5% Low-melt Agarose (Biozym, Oldendorf, Germany). Cells were incubated for 3 days at 37°C and 5% CO_2_ before plaque visualization and counting using 0.1% Neutral Red solution for 3 h (Sigma Aldrich, Missouri, USA).

### Anaphylatoxin Elisa

C3a and C5a levels of plasma samples from COVID-19 patients and healthy donors were detected by the BD OptEIA Human C3a ELISA Kit and BD OptEIA Human C5a ELISA kit respectively (BD Biosciences, Franklin Lakes, NJ, USA) according to the manufacturer’s instructions.

### Statistical Analysis

Statistical analysis was performed using GraphPad Prism 8 software. The differences between negative control and/or different COVID-19 patient groups of ELISpot and flow cytometris analyzes were analyzed using an OneWay ANOVA with Dunnett´s post test. Statistical significance of SARS-CoV-2-specific antibody categories were determined using Kruskal–Wallis and Dunn’s post test (GraphPad Prism). To determine the neutralizing capacity half-maximal neutralizing titer values (IC50 values) from neutralization curves were calculated using four-parameter nonlinear regression and statistical differences were determined using Kruskal–Wallis and Dunn’s post test (GraphPad Prism).

## Results

### Significant High T Lymphocyte Induction in COVID-19 Patients With Mild Disease Progression

**Figure 1:**
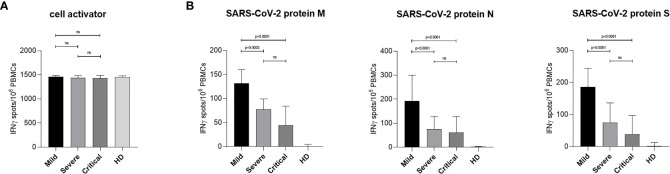
IFNγ production in SARS-CoV-2 specific PBMCs from COVID-19 patients upon activation with virus-specific peptides. PBMCs from COVID-19 patients and HD were isolated from whole EDTA-blood using red blood cell lysis solution. ELISpot Multiscreen^®^ plates were coated overnight with anti-human IFNγ mAb 1-D1K (2 µg/ml). On the following day, PBMCs were stimulated overnight with stimuli as indicated. Spots were revealed by subsequent incubation with biotinylated anti-human IFNγ mAb 7-B6-1 (1 µg/ml), Streptavidin-Alkaline Phosphatase (1:1,000) and ready to use BCIP^®^/NBT. Results are shown as median IFNγ spots normalized by 1 × 10^6^ cells from the different groups, Mild (n = 11), Severe (n = 15), Critical (n = 11), HD (n = 21). Error bars represent interquartile range. **(A)** IFNγ ELISpots of patient PBMCs stimulated with a cell activator cocktail (1:500) are shown. **(B)** IFNγ ELISpots of patient PBMCs stimulated using peptide pools covering the complete SARS-CoV-2 proteins M (left), N (middle) or S (right) are presented. Statistical differences were determined using one-way ANOVA with Dunnett´s post test. ns, not significant.

In order to monitor SARS-CoV-2 specific T cells in COVID-19 patients, we collected peripheral blood from 37 hospitalized individuals with mild (n = 11), severe (n = 15) and critical (n = 11) disease progression. Patients were classified by severity according to ECDC guidelines. Demographic, clinical as well as serological information is summarized in **Table 1** (28). The median age of all patients was 66 years (28–88 years) and the percentage of female and male patients included in the study was 34.4 and 65.6% respectively (**Table 1** **)**. The average sampling day of analyzed samples, defined as the time (days) between positive SARS-CoV-2 PCR testing and blood sampling, was 37 days (**Table 1**). To study T cell responses in COVID-19 patients interferon gamma (IFNγ) ELISpot analyses were performed using commercially available peptide pools of matrix (M) and nucleocapsid (N) protein along with the immunodominant spike (S) protein of SARS-CoV-2 for stimulation of patient cells. Peptide pools consisting of 15-mer sequences with 11 amino acids overlap were used and designed to cover the complete sequences of M, N and S proteins of SARS-CoV-2. Peripheral blood mononuclear cells (PBMCs) were stimulated with peptide pools or with a commercially available cell activation cocktail to assess functionality of same T cell numbers within the various groups. IFNγ spots were counted after 24 h. In **Figure 1A** results from patients with mild, severe and critical COVID-19 together with healthy donors (HD) stimulated with cell activation cocktail are shown. In all tested COVID-19 patients as well as in HD similar amounts of IFNγ spots were detected following unspecific stimulation, indicating that T cell functionality is not impaired due to COVID-19 (**Figure 1A**). Next we investigated activation of SARS-CoV-2 specific T cells by stimulation of PBMCs with peptide pools of M (**Figure 1B**, left), N (**Figure 1B**, middle) and S (**Figure 1B**, right) protein of SARS-CoV-2. Patients with mild COVID-19 symptoms showed significantly higher amounts of IFNγ-producing cells compared to individuals with severe or critical diseases (**Figure 1B**). These increased values of SARS-CoV-2 specific T cells in mild patients were observed for all three virus-specific proteins tested (**Figure 1B**). However, the largest proportion of SARS-CoV-2-specific T cells in patients with a mild course recognized peptides from proteins N and S (**Figure 1B**, middle, right). No significant differences in SARS-CoV-2 specific T cell activation were detected between severe and critical COVID-19 patients, but lowest activation was always found in critically ill patient samples (**Figure 1B**). Cells from healthy donors showed only background or no activation upon stimulation with SARS-CoV-2 specific peptide pools. These results clearly demonstrate a significantly higher T lymphocyte induction in COVID-19 patients with a mild disease progression.

### Strong Cytotoxic T Lymphocyte (CTL) and Protein S-Specific T Helper (Th) Cell Induction in Patients With Mild COVID-19

**Figure 2:**
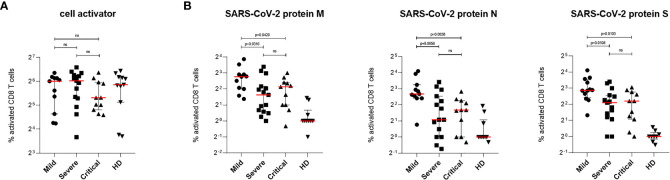
SARS-CoV-2-specific CD8^+^ T cells response in patients with mild, severe or critical COVID-19. PBMCs from COVID-19 patients and HD were stimulated 6 h at 37°C using either cell activator cocktail or SARS-CoV-2 PepTivator^®^ peptide pools. Cells were fixed after extracellular staining for CD8^+^ expression and then permeabilized. Intracellular staining was performed to determine polyfunctionality of T cells by measuring IL-2, IFN-γ, TNF-α and MIP-1β levels. Cells were analyzed by flow cytometry and activation was assessed by comparison of cytokine production with unstimulated cells. T cell activation was increased in all three COVID-19 groups compared to HD group. **(A)** Percentages of activated SARS-CoV-2-specific CD8^+^ T cells upon stimulation with cell activator cocktail are shown for each donor and median values are represented as red lines. **(B)** Percentages of SARS-CoV-2-specific CD8^+^ T cells upon stimulation with peptide pools covering the complete SARS-CoV-2 proteins M (left), N (middle) or S (right) are illustrated. Statistical variability is presented as interquartile range. Statistical differences were determined using the two-tailed unpaired student’s t-test. ns, not significant.

Next, we characterized the distribution of total IFNγ-producing T cells found in ELISpot analyses between CD8^+^ and CD4^+^ T cells by multi-parameter flow cytometry. For this, PBMCs isolated from COVID-19 patients with mild, severe and critical course along with cells from HD were stimulated for 6 h using either SARS-CoV-2-specific peptide pools or cell activator cocktail. CD8^+^ and CD4^+^ T lymphocytes were analyzed by flow cytometry following intracellular staining of interleukin 2 (IL-2), IFNγ, tumor necrosis factor alpha (TNFα) and macrophage inhibitory protein 1 beta (MIP-1β) to investigate activation and polyfunctionality of T lymphocytes. After incubation with cell activator cocktail no significant differences in CD8^+^ T cell activation between patients with mild, severe and critical COVID-19 as well as HD could be detected (**Figure 2A**). **Figure 2B** shows CTL activation after stimulation with SARS-CoV-2 specific protein M (left), N (middle) or S (right). We found that patients with mild COVID-19 showed significantly higher numbers of activated CTLs compared to individuals with severe or critical disease (**Figure 2B**). These significantly higher numbers of SARS-CoV-2 specific T cells in mild patients were observed for all three virus-specific proteins tested (**Figure 2B**). However, the largest proportion of SARS-CoV-2 specific T cells in patients with a mild course recognized peptides from proteins M and N (**Figure 1B**, middle, right).

**Figure 3:**
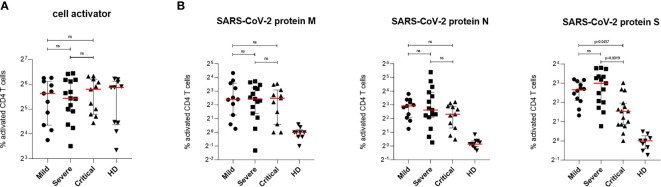
SARS-CoV-2-specific CD4^+^ T cells response in patients with mild, severe or critical COVID-19. PBMCs from COVID-19 patients and HD were stimulated for 6 h at 37°C using either cell activator cocktail or SARS-CoV-2 PepTivator^®^ peptide pools. Cells were fixed after extracellular staining for CD4^+^ expression and then permeabilized. Intracellular staining was performed to determine polyfunctionality of T cells by measuring IL-2, IFN-γ, TNF-α and MIP-1β levels. Cells were analyzed by flow cytometry and activation was assessed by comparison of cytokine production with unstimulated cells. Polyfunctionality of T cells was increased in all three COVID-19 groups compared to HD group. **(A)** Percentages of activated SARS-CoV-2-specific CD4^+^ T cells upon stimulation with cell activator cocktail are shown for each donor and medians are represented as red lines. **(B)** Percentages of SARS-CoV-2-specific CD4^+^ T cells upon stimulation with peptide pools covering the complete SARS-CoV-2 proteins M (left), N (middle) or S (right) are illustrated. Statistical variability is presented as interquartile range. Statistical differences were determined using two-tailed unpaired student’s t-test. ns, not significant.

Similar to CD8^+^ T cell activation upon stimulation with cell activator cocktail, no significant differences were observed when analyzing activation of the CD4^+^ T cell fraction in all patient PBMCs (**Figure 3A**). Stimulation of CD4^+^ T lymphocytes with protein M or N of SARS-CoV-2 revealed no significant changes in cell activation between patients with mild, severe or critical COVID-19 (**Figure 3B**). In contrast, stimulation with protein S showed a significantly higher activation of SARS-CoV-2 specific T cells in patients with mild compared to patients with critical COVID-19 as well as between patients with severe and critical disease progression (**Figure 3B**, right). No significant differences in SARS-CoV-2-specific T cell activation were found between patients with mild and severe COVID-19 (**Figure 3B**, right). Cells from healthy donors showed only background or no activation upon stimulation with SARS-CoV-2 specific peptide pools (**Figure 3B**). Data presented here show strongest induction of SARS-CoV-2-specific CTLs against all tested viral proteins in patients with mild COVID-19. In addition, differences in Th cell activation were only detected against protein S of SARS-CoV-2 revealing lowest T cell activation in patients with critical COVID-19 (**Figure 3B**, right).

### High SARS-CoV-2-Specific Antibody Titers Detected in Patients With Critical COVID-19

**Figure 4:**
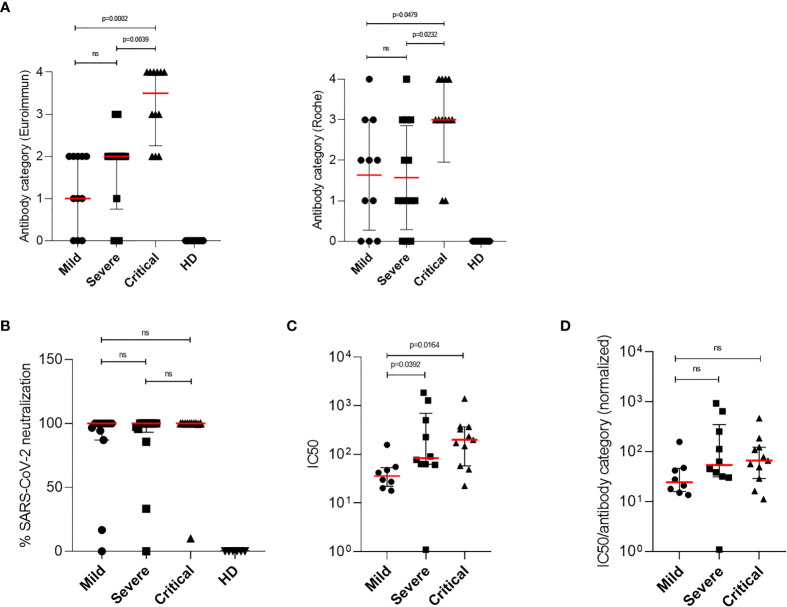
Detection of SARS-CoV-2-specific antibodies and characterization of the neutralizing ability by patient antibodies. Titers of SARS-CoV-2-specific IgG antibodies were determined from plasma samples of COVID-19 patients. **(A)** Antibodies ratios were grouped in four categories (quartiles) and assigned to an antibody category (1 = low, 2 = medium low, 3 = medium high and 4 = high IgG titer). Antibody categories of all mild, severe and critically ill patients and healthy donors, analyzed by the Euroimmun (left) or Roche test kit, are shown. **(B)** Neutralization plaque assays were performed by 1 h incubation of SARS-CoV-2 strains and 1:10 diluted plasma samples from COVID-19 patients or culture medium as control. After incubation plasma–virus mix was ultracentrifuged and resuspended in reduced culture medium and used for inoculation of VeroE6/TMPRSS2 cells. After 1 h inoculation, solution was removed and cells were overlayed with culture medium containing agarose. After 3 days plaques were counted and plaque forming units per ml (PFU/ml) were calculated. Neutralizing ability of patients plasma samples were assessed by the reduction of PFU/ml and compared to medium-treated virus controls. Percentages of SARS-CoV-2 neutralization using a 1:10 dilution of plasma samples from mild, severe and critical COVID-19 patients are shown. **(C)** Neutralization plaque assay over a 1:10 to 1:2,500 dilution range was performed using only IgG positive plasma samples of COVID-19 patients. To determine the neutralizing capacity half-maximal neutralizing titer values (IC50 values) from neutralization curves were calculated using four-parameter nonlinear regression. IC50 values from mild (n = 5), severe (n = 9) and critical patients (n = 11) are illustrated. **(D)** Normalized IC50 values were calculated using the IC50 values from neutralization curves and previously established SARS-CoV-2-specific IgG antibody category from the Euroimmun test kit, due to its specificity towards the viral spike protein. Normalized IC50 values from mild (n = 5), severe (n = 9) and critical (n = 11) COVID-19 patients are shown. Median levels of antibody categories, normalization ability, IC50 and normalized IC50 are represented as red lines and error bars represent interquartile range. Statistical differences were determined using Kruskal–Wallis and Dunn’s post tests. ns, not significant.

To investigate whether antibody production correlates with mild disease progression, virus-specific IgG titers from each patient were measured in plasma samples using two commercially available assays: ELISA testing for S-specific IgG and ECLIA testing for N-specific IgG. SARS-CoV-2 S-specific IgG as well as N-specific IgG titers were classified in four groups (quartiles) and assigned to an antibody category (1 = low, 2 = medium low, 3 = medium high, and 4 = high IgG titer) to enable comparison of the two test systems and calculation of statistical medians of SARS-CoV-2 IgG titers. Although differences between the two test systems could be found, both assays revealed significantly higher SARS-CoV-2-specific IgGs in patients with critical compared to patients with severe or mild diseases [**Table 2** and **Figure 4A** (left, Euroimmun), **Figure 4A** (right, Roche)]. All HD tested negative for SARS-CoV-2 specific IgG antibodies. Among all COVID-19 patients, six plasma samples from patients with mild and severe COVID-19 were negative for S- and N-specific IgGs. These samples were additionally tested by a third commercially available ELISA system, which detects total immunoglobulins directed towards SARS-CoV-2 receptor binding domain (RBD). With this analysis we detected SARS-CoV-2 antibodies in two more COVID-19 patients, whereas the other four patients remained negative for any SARS-CoV-2-specific antibodies (**Table 2**). By using two separate commercially available IgG detection systems we identified higher SARS-CoV-2 S- and N-specific antibody titers in patients with critical COVID-19.

**Table 2:** SARS-CoV-2-specific antibodies evaluated from patients with mild, severe and critical COVID-19.

ID	Severity	IgG (Euroimmun)			IgG (Roche)			Total Ig (Wantai)	
		Results	Ratio	Abs category	Results	Ratio	Abs category	Results	Ratio
1	Mild	Positive	6.5	2	Positive	47.4	2	NA	-
20	Mild	Negative	0.2	0	Negative	0.1	0	Negative	-
27	Mild	Positive	2.7	1	Positive	49	2	NA	-
28	Mild	Positive	5.3	2	Positive	71.3	3	NA	-
37	Mild	Negative	0.2	0	Negative	0.1	0	Negative	-
54	Mild	Positive	2.2	1	Positive	58	3	NA	-
55	Mild	Positive	4.7	2	Positive	7.8	1	NA	-
65	Mild	Positive	5.2	2	Positive	54.1	2	NA	-
66	Mild	Negative	0.5	0	Negative	0.1	0	Positive	17.3
79	Mild	Positive	3.7	1	Positive	104.2	4	NA	-
80	Mild	Negative	0.6	0	Positive	3.9	1	NA	-
2	Severe	Positive	6.8	2	Positive	52	2	NA	-
3	Severe	Positive	5.9	2	Positive	25.5	1	NA	-
7	Severe	Positive	11.62	4	Positive	94.5	4	NA	-
13	Severe	Negative	0.4	0	Negative	0.1	0	Positive	2
15	Severe	Positive	12.4	3	Positive	4.1	1	NA	-
21	Severe	Positive	6.2	2	Positive	71.8	3	NA	-
22	Severe	Negative	0.2	0	Negative	0.1	0	Negative	-
23	Severe	Positive	6.7	2	Positive	17.2	1	NA	-
25	Severe	Positive	6.1	2	Positive	60.8	3	NA	-
26	Severe	Positive	6.5	2	Positive	77.1	3	NA	-
43	Severe	Negative	0.2	0	Negative	0.1	0	Negative	-
44	Severe	Positive	1.2	1	Positive	3.8	1	NA	-
48	Severe	Positive	9.1	3	Positive	7.9	1	NA	-
50	Severe	Positive	6.2	2	Positive	92.4	4	NA	-
57	Severe	Positive	4.5	2	Positive	33.4	1	NA	-
10	Critical	Positive	12.2	4	Positive	53	3	NA	-
11	Critical	Positive	14.4	4	Positive	78.2	4	NA	-
12	Critical	Positive	12.4	4	Positive	86.5	4	NA	-
16	Critical	Positive	9.7	3	Positive	69.7	3	NA	-
17	Critical	Positive	10.2	3	Positive	11.9	1	NA	-
24	Critical	Positive	5.9	2	Positive	104.2	4	NA	-
38	Critical	Positive	6.4	2	Positive	58.8	3	NA	-
39	Critical	Positive	7.2	2	Positive	16.6	1	NA	-
40	Critical	Positive	11.6	4	Positive	81.8	4	NA	-
41	Critical	Positive	14.4	4	Positive	71	3	NA	-
42	Critical	Positive	12.1	4	Positive	67.3	3	NA	-

Antibody ratios of all 37 patients diagnosed with COVID-19 analyzed by three SARS-CoV-2-specific test kits (Euroimmun, Roche and Wantai) are shown.

### Potent Neutralization Detected in All SARS-CoV-2 Seropositive COVID-19 Patients

In a next step we determined the overall level of neutralizing activity in the cohort. For this we performed neutralization assays using a range of various dilutions (1:10 to 1:2,500) of plasma samples from COVID-19 patients with mild, severe and critical symptoms. In all plasma samples tested positive for SARS-CoV-2-specific IgG antibodies, a neutralizing effect was illustrated at the lowest dilution (1:10, **Figure 4B**). To further investigate the neutralizing capacity, half-maximal neutralizing titer values (IC50 values) from neutralization curves were calculated. For this, neutralization assays over a 1:10 to 1:2,500 dilution range were performed from plasma samples of COVID-19 patients with mild, severe and critical disease progression (**Supplementary Figure 1**). IC50 values were calculated by nonlinear regression and results are shown in **Figure 4C**. These analyses exhibited that significantly higher IC50 values were detected in patients with critical compared to mild COVID-19 (**Figure 4C**). Even though elevated IC50 levels were also determined in patients with severe compared to mild COVID-19, these differences were not significant (**Figure 4C**). Results from antibody titers and neutralization assays (IC50 values) observed in severe and critical COVID-19 patients suggest that there is a correlation between higher amounts of antibodies and enhanced neutralization of SARS-CoV-2. Therefore we next performed correlation analyses between antibody titers and corresponding IC50 values from each patient using two-tailed nonparametric Spearman correlation. Overall, we found a significant, positive and moderate relation (r = 0.5084; p = 0.0041) for all patients groups, which indicates that calculated IC50 values without further correction do not allow a comparison of patients with mild, severe and critical COVID-19 (**Supplementary Figure 2**). Thus, we next normalized IC50 values with the previously established SARS-CoV-2-specific IgG antibody categories obtained from the Euroimmun test kit, due to its specificity towards the viral spike protein. These analyses exhibited that no significant differences in the neutralizing capacity of SARS-CoV-2-specific antibodies from patients with mild, severe and critical COVID-19 were detected (**Figure 4D**). By determining normalized IC50 values we could show that potent neutralizing antibodies were found in COVID-19 patients irrespective of their disease progression.

### Elevated Anaphylatoxin C3a and C5a Levels Detected in Severe and Critical COVID-19 Patients

**Figure 5:**
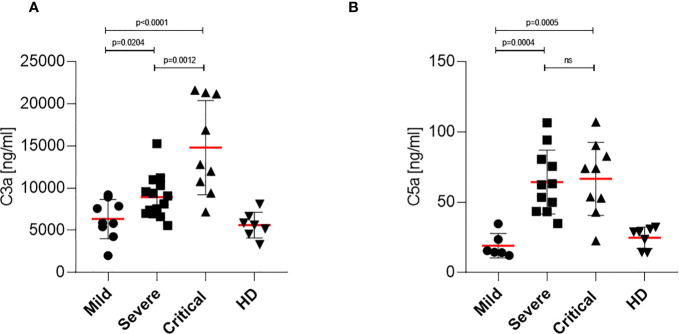
Determination of anaphylatoxin levels in COVID-19 patients. **(A)** C3a level determination in mild, severe and critical COVID-19 patients as well as healthy donors. Plasma samples were harvested and C3a levels were analyzed using a BD Biosciences OptEIA Human C3a ELISA kit. **(B)** C5a level determination in mild, severe and critical COVID-19 patients as well as healthy donors. Plasma samples were harvested and C5a levels were analyzed using a BD Biosciences OptEIA Human C5a ELISA kit. C3a and C5a levels in ng/ml were determined for all patients and healthy donors and plotted on a bar graph. ns, not significant.

Since it has been reported that in severe and critical COVID-19 patients high anaphylatoxin serum levels were detected and because we found high antibody titers in patients that could potentially activate complement, we also investigated C3a and C5a plasma levels of patients with mild, severe and critical COVID-19. C3a and C5a levels of plasma samples were analyzed using commercially available ELISA kits and results are illustrated in **Figures 5A, B**, respectively. Significantly higher levels of the anaphylatoxin C3a were detected in plasma samples of severe and critical COVID-19 patients, with the highest levels found in patients with critical symptoms (**Figure 5A**). In contrast, only low or background levels of C3a were measured in patient samples with mild COVID-19, which corresponded to the plasma levels of HD (**Figure 5A**). Expression of the anaphylatoxin C5a was similar and significantly increased in patient samples of severe and critical COVID-19 compared to plasma samples of patients with mild symptoms. In addition, expression of C5a was found lowest in plasma samples of mild COVID-19 patients and comparable to the values detected in healthy donors (**Figure 5B**). These data revealed an overshooting anaphylatoxin response in severe and critical COVID-19 patients. Notably, COVID-19 patients with mild symptoms showed no elevated levels of the anaphylatoxins C3a and C5a.

## Discussion

T cells play a fundamental role in the early control and clearance of many viral infections of the respiratory system (29). Also in SARS-CoV-2 infected individuals, lymphopenia with drastically reduced CD4^+^ and CD8^+^ T cells correlates with COVID-19-associated disease severity and mortality (30). A specific feature for SARS-CoV-2 infection with a high impact on disease progression is represented by the cytokine profile induced in acute infection (31). In fact, a maladapted immune response profile with strong pro-inflammatory and decreased tissue reparative growth factors were associated with severe COVID-19 and poor clinical outcome (31). In this context, we and others identified a link between complement regulation and cytokine overexpression in SARS-CoV-2 infected tissues, since complement deposition and high anaphylatoxin serum levels have been reported in patients with severe or critical COVID-19 (25). Here we analyzed T cell immunity of COVID-19 patients with mild, severe and critical disease progression using peptide pools for three major immunodominant viral proteins. Independent characterization between SARS-CoV-2-specific CD4^+^ and CD8^+^ T lymphocytes and furthermore data on the antigen specificity of the T cells were obtained. In addition to T cell immunity also S- and N-specific IgG antibody titers as well as assays to study the neutralization capacity of antibodies detected in patient plasma samples were performed. Finally, we investigated the anaphylatoxin levels in plasma samples of COVID-19 patients regarding disease progression.

We found that COVID-19 patients with mild disease progression show significantly higher T lymphocyte induction compared to patients with severe or critical symptoms. Furthermore, we demonstrated that T cells recognized all three tested SARS-CoV-2 peptide pools, but the largest proportion of SARS-CoV-2 specific T cells in patients with a mild course recognized peptides from protein N and protein S. These findings are in accordance to the literature, since Ni and colleagues previously reported that mild COVID-19 patients showed a robust T cell response for viral M, N and S proteins, which subsequently shifted to only N-specific T cells in about one-third of the cases post recovery (18). Another study investigated virus-specific CD4^+^ and CD8^+^ T cells of 10 COVID-19 patients with severe ARDS. By flow cytometry they found in 10 out of 10 and in eight out of 10 patients that CD4^+^ and CD8^+^ cells respectively showed the strongest T cell responses directed to the viral spike glycoprotein (32). More recently, Dan et al. investigated memory T cells in a cohort of COVID-19 cases with broad range of severity and found 70% of subjects had SARS-CoV-2 memory CD8^+^ T cell and 93% had memory CD4^+^ T cell 1 month post-symptoms onset (20–50 days) (17). In accordance to this, flow cytometric analyses of our patient cohort revealed that 33 out of 37 (89%) patients had S-specific CD8^+^ T cell while 35 out of 37 (95%) patients had S-specific CD4^+^ T cell. All patients with no specific T cells were classified within severe or critical disease groups. In contrast, the highest T cell activation was not only directed to viral protein S, but also to protein N. Highest numbers of SARS-CoV-2-specific CD8^+^ T cells recognizing M, N, and S protein were consistently found in patients with mild COVID-19. Furthermore, critical COVID-19 patients showed the lowest activation of S protein-specific CD4^+^ T cells, while similar activation compared to patients with mild and severe symptoms was detected for CD4^+^ T cells directed to M or N viral protein. Due to the investigation of early samples from COVID-19 patients here, this might explain the reduced frequencies of SARS-CoV-2-specific CD4^+^ T cells in severe patients, which was not observed in other studies on recovered COVID-19 patients (17, 33). Interestingly, Peng et al. also reported that the breadth and magnitude of T cell immunity is greater in more severe cases of COVID-19, but at the same time the proportion of activated and proliferating CD8^+^ T cells is increased in patients with mild COVID-19 (20, 33). As also illustrated here, we characterized the highly abundant SARS-CoV-2-specific CD8^+^ T cell response and furthermore we demonstrated the extensive polyfunctionality of these T cells upon stimulation, similar to Sekine et al. (20). In addition to T cell immunity we here also investigated humoral immune responses and analyzed the presence of SARS-CoV-2-specific IgG antibodies against S- and N-proteins. In general antibody titers are used to assess protection, but here we found the highest titers of SARS-CoV-2 antibodies were found in patients with critical COVID-19, which indicates that a robust antibody response alone is insufficient to avoid severe disease. This is in line with previous data, since SARS-CoV and SARS-CoV-2 high antibody titers and early seroconversion were reported as indication of severe disease progression (34–38). Despite these findings, virus-specific IgM, IgG, IgA and neutralizing IgG antibodies are induced after seroconversion, which occurs in most COVID-19 patients between 7 and 14 days after onset of symptoms (30). In a next step we determined the neutralizing abilities of antibodies detected in COVID-19 patient samples based on half-maximal neutralizing responses (IC50 values) of neutralization curves. No significant differences in viral neutralization could be observed between patients with mild, severe or critical COVID-19. Antibody responses against SARS-CoV-2 have been the focus of many investigations and these studies revealed that mostly antibodies directed to internal N or external S protein were detected. In addition, potentially neutralizing antibodies recognized the receptor binding domain (RBD) and may thereby block virus and cell receptor interactions (39, 40). Non-neutralizing virus-specific IgG antibodies were previously reported to contribute to pulmonary pathology by facilitating viral entry to Fc-receptor expressing cells, such as dendritic cells, macrophages and monocytes (41, 42). Enhanced uptake of viral particles was associated in some cases with increased inflammatory responses or with impaired induction of virus-specific cytotoxic T lymphocytes (41, 43). *In vitro* experiments additionally revealed that serum containing antibodies against the S protein from SARS-CoV patients enhanced infection in human monocyte-derived macrophages, which might also happen in SARS-CoV-2 (42). Therefore, the increased levels of SARS-CoV-2 specific antibodies, in combination with elevated anaphylatoxin levels found here further corroborate this aspect. Although, no evidence for such pathological features have been reported in COVID-19 patients so far, the possibility of antibody-dependent enhancement (ADE) has to be considered in the developments of vaccination or therapeutical approaches. Other studies speculate that ADE exists in SARS-CoV-2 and that suboptimal antibody responses represents a potential danger in COVID-19 patients, since also neutralizing antibodies can interact with other immune components, such as complement, phagocytes and natural killer cells (44, 45). Recently, it was also demonstrated that enhanced ADE in *in vitro* experiments using monoclonal patient antibodies targeting SARS-CoV-2 RBD epitopes (46). In most cases, these interactions of various immune components enhance viral clearance but pathogen-specific antibodies might on the other hand promote ADE (44).

Additionally, complement proteins such as anaphylatoxins were reported to be also produced and secreted by epithelial cells and additionally were found to be highly detected in serum of patients with severe or critical COVID-19 (25). Normally, complement should be protective during viral infections, but with respect to COVID-19, local complement activation might worsen tissue injury (47, 48). Here we demonstrated that both anaphylatoxins C3a and C5a were highly detectable in patient plasma with severe or critical COVID-19, which was previously associated with worsening of injury in the airway epithelium and with inflammasome activation in immune cells and lung tissues (49, 50). Moreover a study by Cugno et al. also correlated high C5a and soluble component 5b-9 level with COVID-19 severity (51). Activation of systemic complement system by IgG containing immune complexes occur mainly *via* the classical pathway (52). Thus, the detected higher antibody responses in severe and critical patients might cause augmented formation of immune complexes, which subsequently promotes complement activation *via* the classical pathway. This is also supported by clinical studies administering the C3 antagonist AMY-1 or the C5a activation inhibitor eculizumab to severely ill COVID-19 patients, which resulted in disease recovery (38). Furthermore, treatment with monoclonal antibodies against anaphylatoxin C5a receptor 1 (C5aR1) prevented C5a-mediated recruitment and activation of human myeloid cells and inhibited acute lung injury in *in vivo* experiments (47). Indeed, our group demonstrated that targeting receptors for C3a and C5a from non-immune cells prevented lung inflammation and tissue damage in a 3D respiratory tissue model (27).

Here, we specifically investigated T cell immunity, antibody responses and anaphylatoxin levels in patients with mild, severe and critical symptoms of patients with COVID-19. Crucially, we provide a full picture of cellular and humoral immune responses of COVID-19 patients and prove again that robust polyfunctional CD8^+^ T cell responses as well as low anaphylatoxin levels correlate with mild infection. In addition, our data also supports the idea of blocking the C5a–C5aR1 axis as therapeutic strategy to limit excessive lung inflammation and tissue damage caused by infiltrating myeloid cells.

## Data Availability Statement

The original contributions presented in the study are included in the article/**Supplementary Material**. Further inquiries can be directed to the corresponding author.

## Ethics Statement

Written informed consent to participate in this study was obtained from all donors of leftover nasopharyngeal/ oropharyngeal specimens, EDTA blood and Serum samples by the participating clinics. The Ethics Committee of the Medical University of Innsbruck approved the use of anonymized leftover specimens of COVID-19 patients (ECS1166/2020) and healthy donors (ECS1166/2018) for scientific purposes.

## Author Contributions

Conceptualization: CF and WP. Data curation: EL and WP. Formal analysis: EL, GD, CW, MR, AB, and WP. Funding acquisition: DW, CL-F, and WP. Investigation: EL, GD, CW, VZ, AB, GH, CM, and WP. Methodology: EL, GD, CW, RB-W, MR, AG, VF, GH, and WP. Project administration: EL and WP. Resources: MR, AG, CM, AZ, EW, DW, CL-F, and WP. Software: EL, GD, AG, VF, DW, and WP. Supervision: CM, EW, DW, CL-F, and WP. Validation: EL, GD, VZ, RB-W, MR, VF, CM, AZ, EW, and WP. Visualization: DW and WP. Writing—original draft: EL, GD, DW, and WP. Writing—review &amp. editing: EL, GD, CW, VZ, RB-W, MR, AB, AG, VF, GH, CM, AZ, EW, DW, CL-F, and WP. All authors contributed to the article and approved the submitted version.

## Funding

The authors were supported by the Austrian Science Fund (FWF; P 34070-B to WP and P33510-B to DW), the Anniversary Fund of the Austrian National Bank (OeNB; P 17614 to WP and P 17633 to DW) and the State of Tyrol (No. 70454 to WP).

## Conflict of Interest

The authors declare that the research was conducted in the absence of any commercial or financial relationships that could be construed as a potential conflict of interest.
