# Supplementary material for: Potent SARS-CoV-2-Specific T Cell Immunity and Low Anaphylatoxin Levels Correlate With Mild Disease Progression in COVID-19 Patients
Source: Front Immunol. 2021 Jun 14;12:684014. doi: 10.3389/fimmu.2021.684014 (PMC8237940; doi:10.3389/fimmu.2021.684014)
Supplement: Supplementary file 1 [file Presentation_1.pptx]

## Slide 1
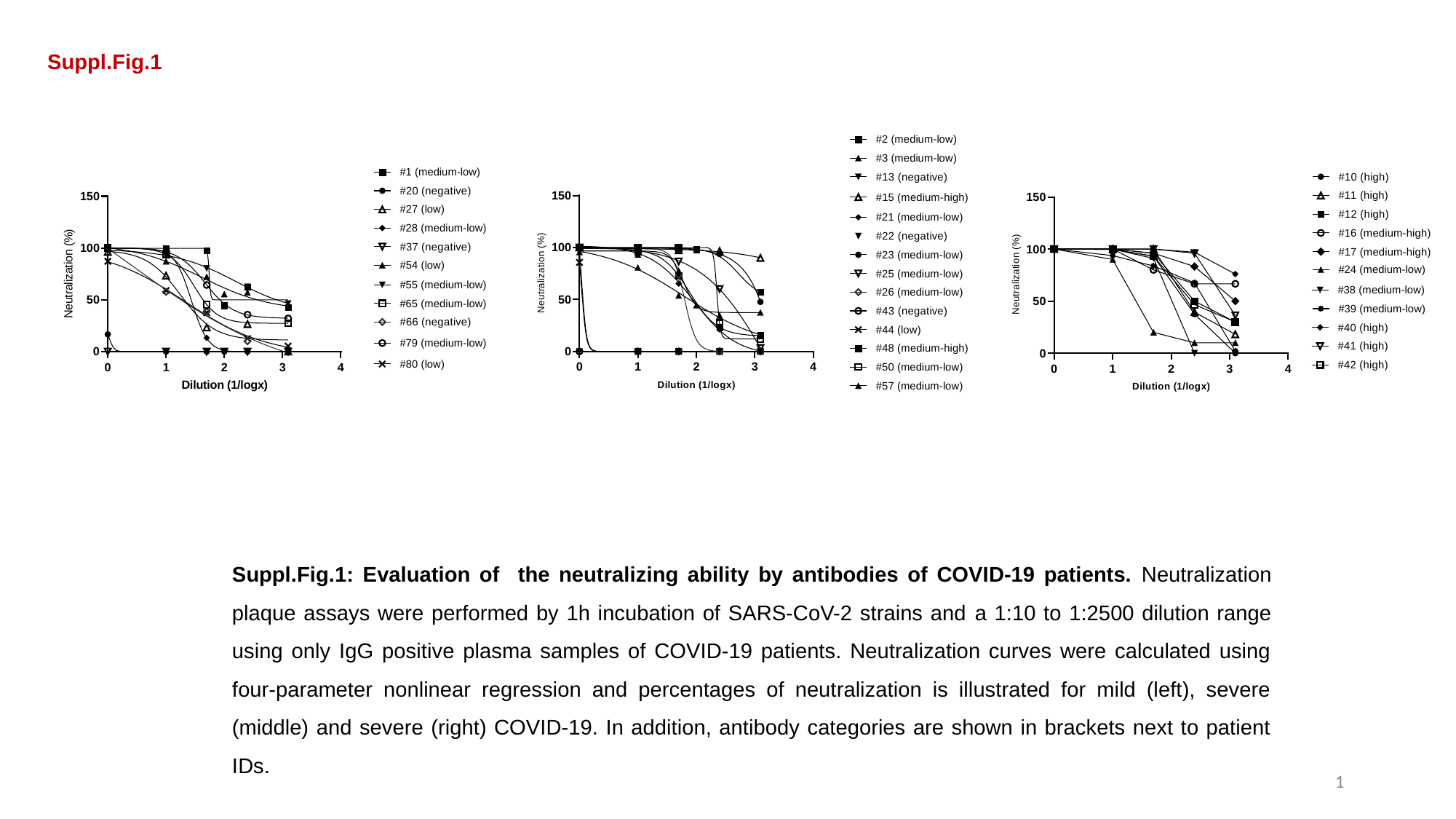

Suppl.Fig.1
Suppl.Fig.1: Evaluation of the neutralizing ability by antibodies of COVID-19 patients. Neutralization plaque assays were performed by 1h incubation of SARS-CoV-2 strains and a 1:10 to 1:2500 dilution range using only IgG positive plasma samples of COVID-19 patients. Neutralization curves were calculated using four-parameter nonlinear regression and percentages of neutralization is illustrated for mild (left), severe (middle) and severe (right) COVID-19. In addition, antibody categories are shown in brackets next to patient IDs.
1

## Slide 2
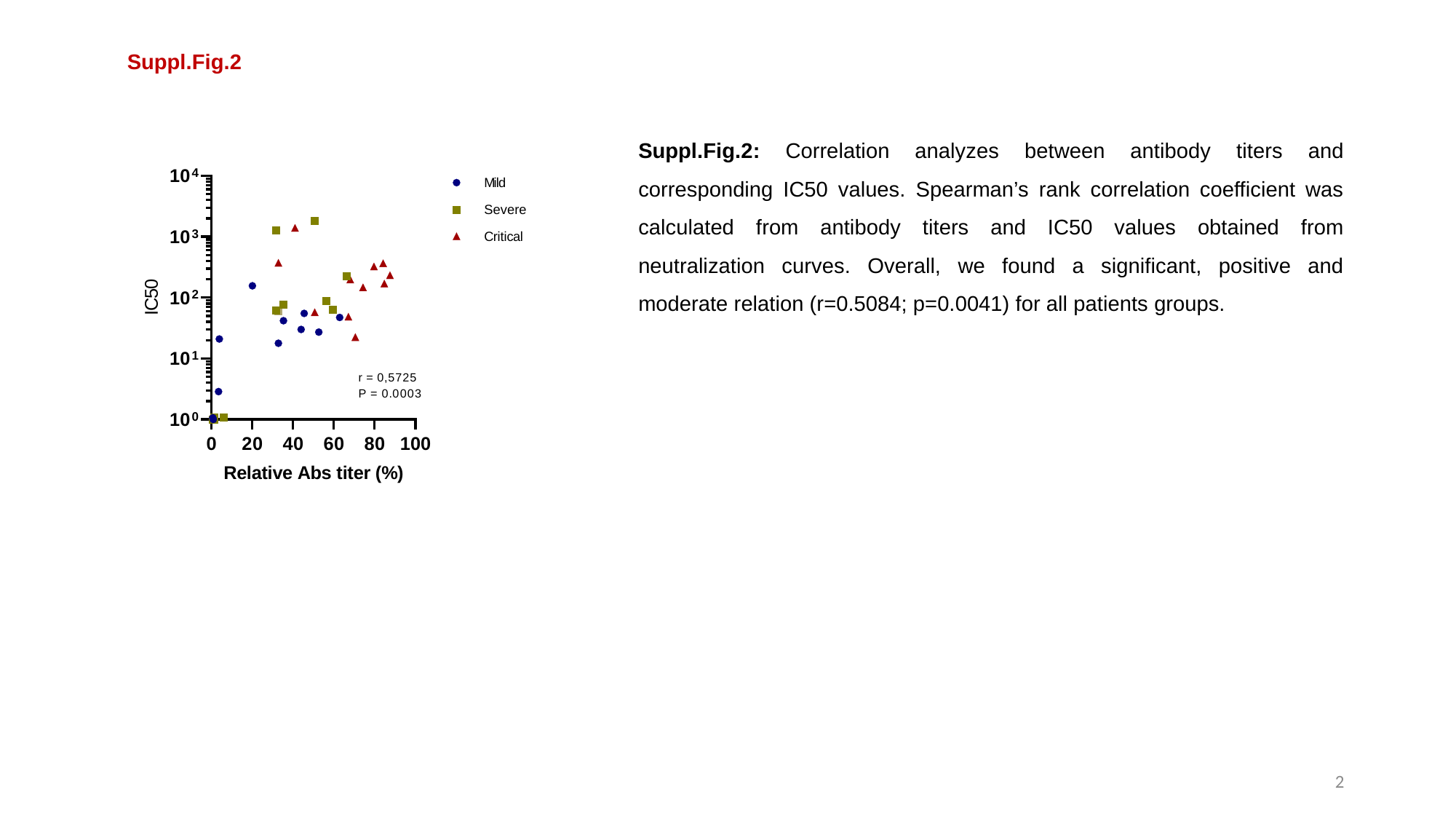

Suppl.Fig.2
Suppl.Fig.2: Correlation analyzes between antibody titers and corresponding IC50 values. Spearman’s rank correlation coefficient was calculated from antibody titers and IC50 values obtained from neutralization curves. Overall, we found a significant, positive and moderate relation (r=0.5084; p=0.0041) for all patients groups.
2
